# A Derived Morphology of the Quadrate May Support a Previously Unrecognized Major Higher‐Level Clade of Neoavian Birds

**DOI:** 10.1002/jmor.70070

**Published:** 2025-07-30

**Authors:** Gerald Mayr

**Affiliations:** ^1^ Senckenberg Research Institute and Natural History Museum Frankfurt Ornithological Section Frankfurt am Main Germany

**Keywords:** anatomy, Aves, osteology, phylogeny, quadratum

## Abstract

The avian quadrate plays a critical role in cranial kinesis, but few comparative studies exist of its morphological variation across higher‐level taxa. The present paper surveys the occurrence of a markedly concave articular facet of the condylus medialis. It is detailed that this feature, for which the term trochlea lateralis is introduced, may represent an apomorphy of a higher‐level clade that includes the Aequornithes (gaviiforms, procellariiforms, suliforms, pelecaniforms, and allies), Phaethontimorphae (tropicbirds, sunbittern, and kagu), Mirandornithes (flamingos and grebes), and Gruiformes (cranes and allies). Like many other morphological characters, the occurrence of the trochlea lateralis shows homoplasy. However, at least one analysis of sequence data found a clade including the aforementioned four taxa, the interrelationships of which are not conclusively resolved in other studies. A trochlea lateralis is present in birds with different cranial morphologies and feeding adaptations, so that its occurrence often seems to have a phylogenetic (shared common ancestry) rather than a functional origin. The morphology of the condylus medialis of the quadrate may also bear on the affinities of some fossil taxa, such as the early Eocene Halcyornithidae and Messelasturidae, in which a trochlea lateralis is present.

## Graphical abstract

The avian quadrate plays a critical role in cranial kinesis, but few comparative studies exist of its morphological variation across higher‐level taxa. In this study, the occurrence of a markedly concave articular facet of the condylus medialis is surveyed across neornithine birds. It is detailed that the feature, for which the term trochlea lateralis is introduced, may represent an apomorphy of a major higher‐level clade.

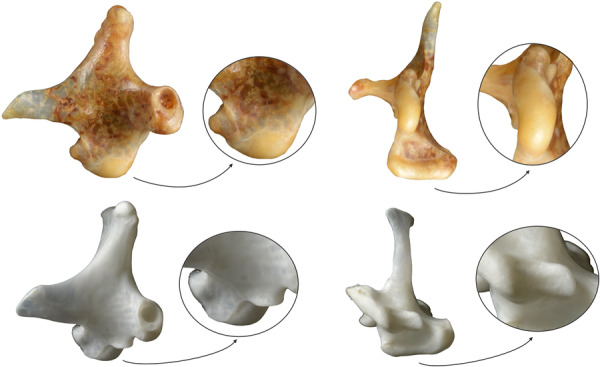

## 1 Introduction

Avian phylogeny has become much better understood in the past decades, but the interrelationships of the higher‐level clades of Neoaves (all extant birds except for the Palaeognathae and Galloanseres) remain largely unresolved. Most current efforts focus on analyses of large genomic data sets, but although these congruently recover certain major neoavian clades, they show conflicting results regarding their relationships relative to each other.

Several of the well‐supported clades were already recognized in a similar form by some 19th century anatomists (Mayr 2011a), but today morphological characters play a subordinate role in attempts to reconstruct neoavian interrelationships. Here, I assess the significance of a feature of the quadrate bone in this regard and hypothesize that it possibly supports a major higher‐level clade.

The avian quadrate plays a critical role in cranial kinesis and shows considerable variation across different clades. Still, there exist few comparative studies across higher‐level taxa. Earlier authors described major features of selected clades (Walker 1888; Lowe 1926; Samejima and Otsuka 1987), but some of the most detailed studies were performed on the quadrates of Mesozoic non‐neornithine avians and galloanserine birds (Elzanowski et al. 2001; Elzanowski and Stidham 2010, 2011; Elzanowski 2013). The quadrate of neoavians remains poorly studied in a comparative context, even though its morphology was described for the Ardeidae (Elzanowski and Zelenkov 2015) and for some upupiform and coraciiform birds (Elzanowski and Boles 2015). 3D geometric morphometric data of the avian quadrate were recently evaluated in broader taxonomic contexts (Kuo et al. 2023, 2024), but few published data exist concerning quadrate morphologies characterizing neoavian higher‐level clades.

One of the most distinctive features of the quadrate of some neoavians is the occurrence of a concave articular facet formed by the lateral portion of the condylus medialis. This facet remained understudied in a phylogenetic context, and published data on its occurrence include some erroneous statements. The feature was referred to as accessory trochlea of the “anterior condyle” by Walker (1888), who first noted its presence in some taxa. It was assessed in a broader comparative context by Bock (1960), who termed it “lateral groove” or “lateral concavity.” Strauch (1978: character 11) and Mayr and Clarke (2003: character 37) included the feature in phylogenetic analyses; the latter mistakenly designated the concavity as “rostrally projecting.” Elzanowski and Boles (2015) and Elzanowski and Zelenkov (2015) referred to the concavity as the trochlea of the medial condyle and commented on its occurrence in the Upupiformes and Ardeidae, respectively.

Inspired by the study of three dimensionally preserved quadrates from the early Eocene British London Clay, I present a survey of the lateral concavity of the condylus medialis and comment on the potential phylogenetic significance of this feature, which shows variation within different clades and can easily be assessed owing to its clear‐cut delimitation.

## 2 Materials and Methods

Quadrates were examined in the skeletal collection of the ornithological section of Senckenberg Research Institute Frankfurt, which includes quadrates of all non‐passeriform family‐level taxa except for the charadriiform Ibidorhynchidae, Pedionomidae, and Pluvianellidae. The examined material stems from the collections of Senckenberg Research Institute and Natural History Museum Frankfurt, Germany (SMF), the Institute of Geosciences of Johannes‐Gutenberg‐University Mainz, Germany (GPIM), and the National Museums Scotland, Edinburgh, UK (NMS).

Ancestral character states were reconstructed with Mesquite 2.71 (Maddison and Maddison 2009).

## 3 Results

**FIGURE 1:**
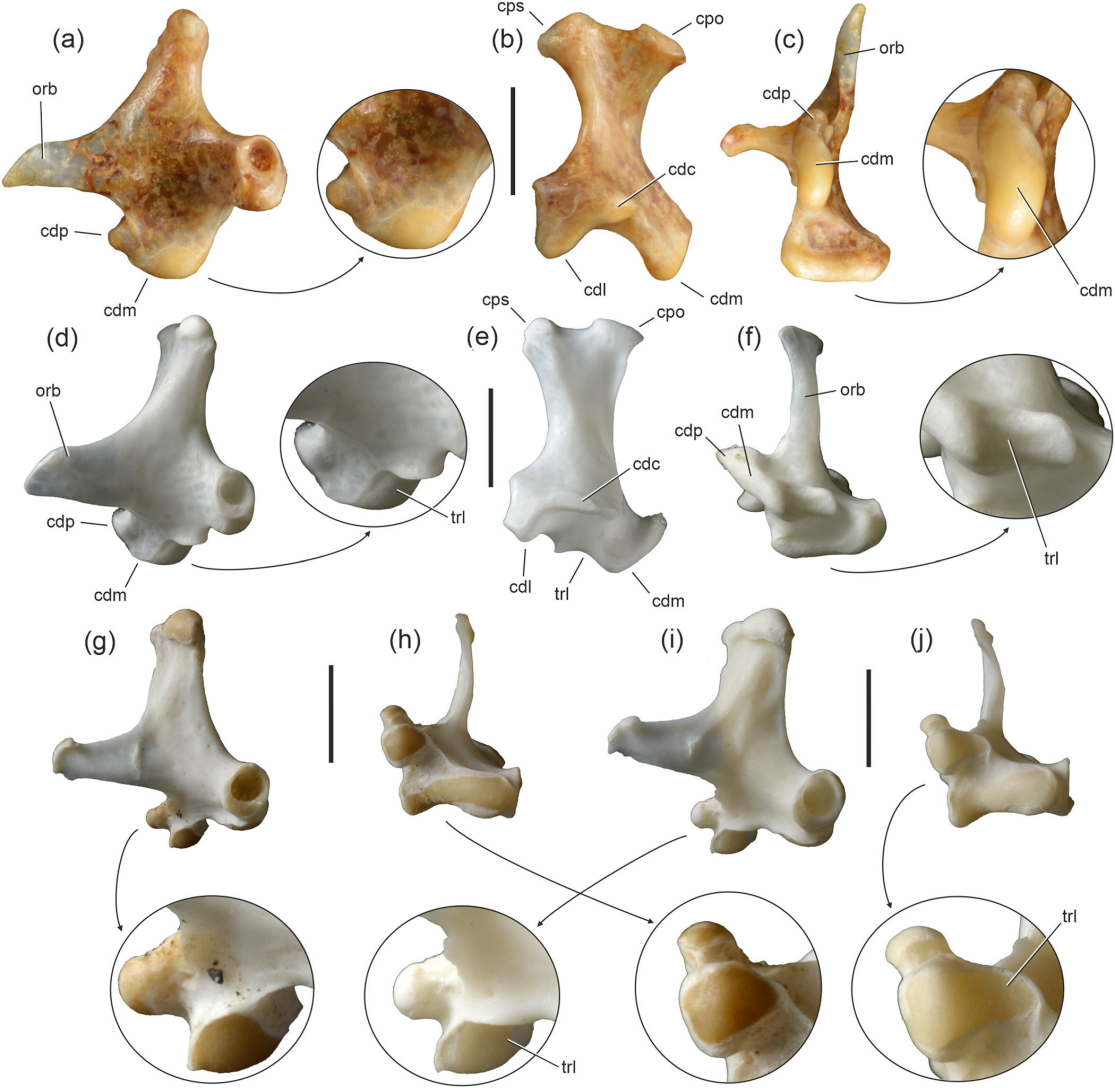
Quadrate morphologies of species and specimens without (a‒c, g, h) and with (d‒f, i, j) a trochlea lateralis; the arrows indicate enlarged details of the condylus medialis. (a‒c) left quadrate of *Leptosomus discolor* (Leptosomidae; SMF 5438) in lateral (a), caudal (b), and ventral (c) view; this species lacks a trochlea lateralis. (d‒f) left quadrate of *Scopus umbretta* (Scopidae; SMF 16592) in lateral (d), caudal (e), and ventral (f) view; this species has a distinct trochlea lateralis. (g‒j) left quadrates of *Alca torda* (Alcidae) in lateral (g, i) and ventral (h, j) view; the specimen in (g) and (h) (SMF 13990) lacks a trochlea lateralis, whereas the trochlea lateralis is present in the specimen in (i) and (j) (SMF 13988). Abbreviations: cdc, condylus caudalis; cdl, condylus lateralis; cdm, condylus medialis; cdp, condylus pterygoideus; cpo, capitulum oticum; cps, capitulum squamosum; orb, processus orbitalis; trl, trochlea lateralis. The scale bar equals 5 mm.

The condylus medialis usually has an ovoid shape and a convex surface (Figure 1a–c), which articulates with the cotyla medialis of the mandible. In some taxa, its lateral portion exhibits a shallow dimple (e.g., Otidiformes as well as some Charadriiformes, Strigidae, and Alcedinidae) or a lateral roughness (e.g., many Palaeognathae). However, a distinct, concave articular facet (Figure 1d–f), which abuts the tuberculum intercotylare of the mandible, is restricted to only a few neoavian higher‐level clades that are surveyed in the following. The lateral concavity of the condylus medialis has been referred to as a trochlea by some authors (Walker 1888; Elzanowski and Zelenkov 2015) and is termed trochlea lateralis in the following.

### 3.1 Aequornithes

**FIGURE 2:**
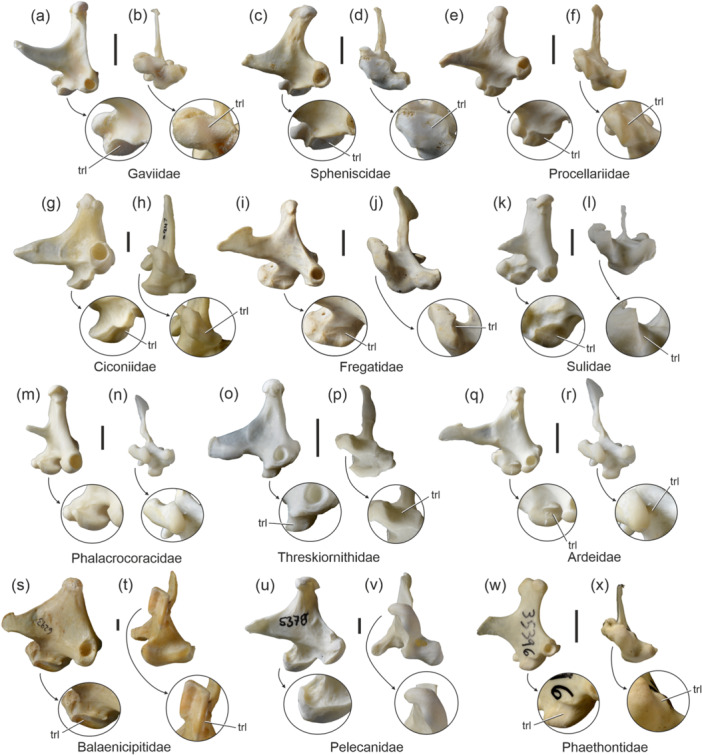
Left quadrate of the Aequornithes and Phaethontiformes in lateral (left) and ventral (right) view; the arrows indicate enlarged details of the condylus medialis. (a, b) *Gavia stellata* (Gaviiformes; SMF 14210). (c, d) *Megadyptes antipodes* (Sphenisciformes; SMF 22581). (e, f) *Procellaria aequinoctialis* (Procellariiformes; SMF 19221). (g, h) *Mycteria leucocephala* (Ciconiiformes; SMF 2847). (i, j) *Fregata magnificens* (Fregatidae; SMF 5389). (k, l) *Sula variegata* (Sulidae; SMF 13641). (m), (n) *Phalacrocorax carbo* (Phalacrocoracidae; SMF 13660); this species lacks a trochlea lateralis. (o), (p) *Geronticus eremita* (Threskiornithidae; SMF 13341). (q, r) *Nycticorax nycticorax* (Ardeidae; SMF 13330). (s, t) *Balaeniceps rex* (Balaenicipitidae; SMF 6293). (u, v) *Pelecanus onocrotalus* (Pelecanidae; SMF 5378); this species lacks a trochlea lateralis. (w, x) *Phaethon lepturus* (Phaethontidae; SMF 9696). Abbreviations: trl, trochlea lateralis. The scale bar equals 5 mm.

Aequornithes is the clade, which includes the Gaviiformes, Sphenisciformes, Procellariiformes, as well as the taxa of the traditional “Ciconiiformes” and “Pelecaniformes” except for the Phaethontidae (Mayr 2011a). A marked and mediolaterally extensive trochlea lateralis is present in the Gaviiformes (Figure 2a,b; including early Eocene stem group representatives; Mayr and Kitchener 2022), Sphenisciformes (Figure 2c,d; including Paleocene stem group representatives; Mayr et al. 2025), Procellariiformes (Figure 2e,f), Ciconiiformes (Figure 2g,h), Threskiornithidae (Figure 2o,p; including early Eocene stem group representatives; Mayr and Kitchener 2023), and Scopidae (Figure 1d–f). In the Ardeidae (Figure 2q,r), the trochlea lateralis is situated in a sulcus between the ventrally prominent condylus medialis and condylus lateralis (see also Elzanowski and Zelenkov 2015). Within the Suliformes, only the Sulidae exhibit a distinct trochlea lateralis (Figure 2k,l). This concavity is absent in the Phalacrocoracoidea (Phalacrocoracidae and Anhingidae; Figure 2m,n), in which the condylus medialis forms a laterally overhanging lip and a lateral roughness demarks the area of the trochlea lateralis in other suliform birds. In the Fregatidae (Figure 2i,j) the trochlea lateralis is shallow (Bock 1960: 41 described it as an “overhanging anterior lip”). The quadrate of the Pelecanidae (Figure 2u,v) lacks a well‐delimited trochlea lateralis, but the lateral surface of the condylus medialis exhibits an irregularly textured surface that is ventrally bordered by a sharp rim formed by the margin of the articular facet. In the Balaenicipitidae (Figure 2s,t), which may (Prum et al. 2015) or may not (Hackett et al. 2008; Kuhl et al. 2021) be the sister taxon of the Pelecanidae, the trochlea lateralis is dorsoventrally narrow.

**FIGURE 3:**
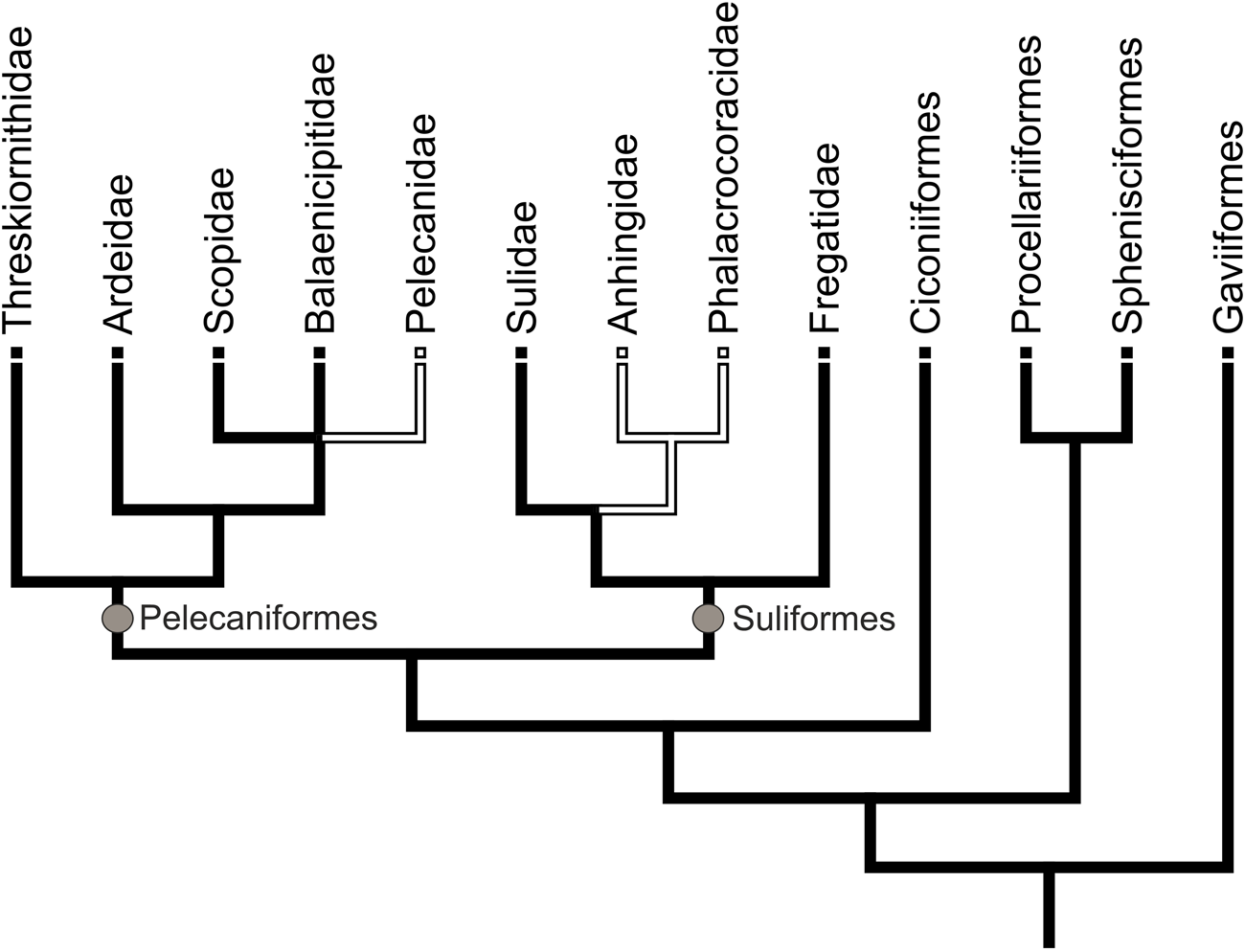
Ancestral state reconstruction of the distribution of a concave lateral facet of the condylus medialis of the quadrate in taxa of the Aequornithes based on the phylogenies of Prum et al. (2015) and Kuhl et al. (2021), which differ in the position of the Balaenicipitidae. Solid black lines indicate presence of the character, black outlined lines its absence.

Ancestral state reconstruction suggests that the trochlea lateralis was present in the stem species of the Aequornithes and was reduced in the Phalacrocoracoidea and Pelecanidae (Figure 3).

### 3.2 Phaethontimorphae

The Phaethontimorphae (sensu Sangster et al. 2022) is a recently proposed taxon for a clade including the Phaethontiformes and Eurypygiformes, which so far only received molecular support. All representatives of this clade exhibit a trochlea lateralis, which is less pronounced in the Phaethontiformes (Figure 2w,x) and Rhynochetidae than in the Eurypygidae.

### 3.3 Gruiformes

**FIGURE 4:**
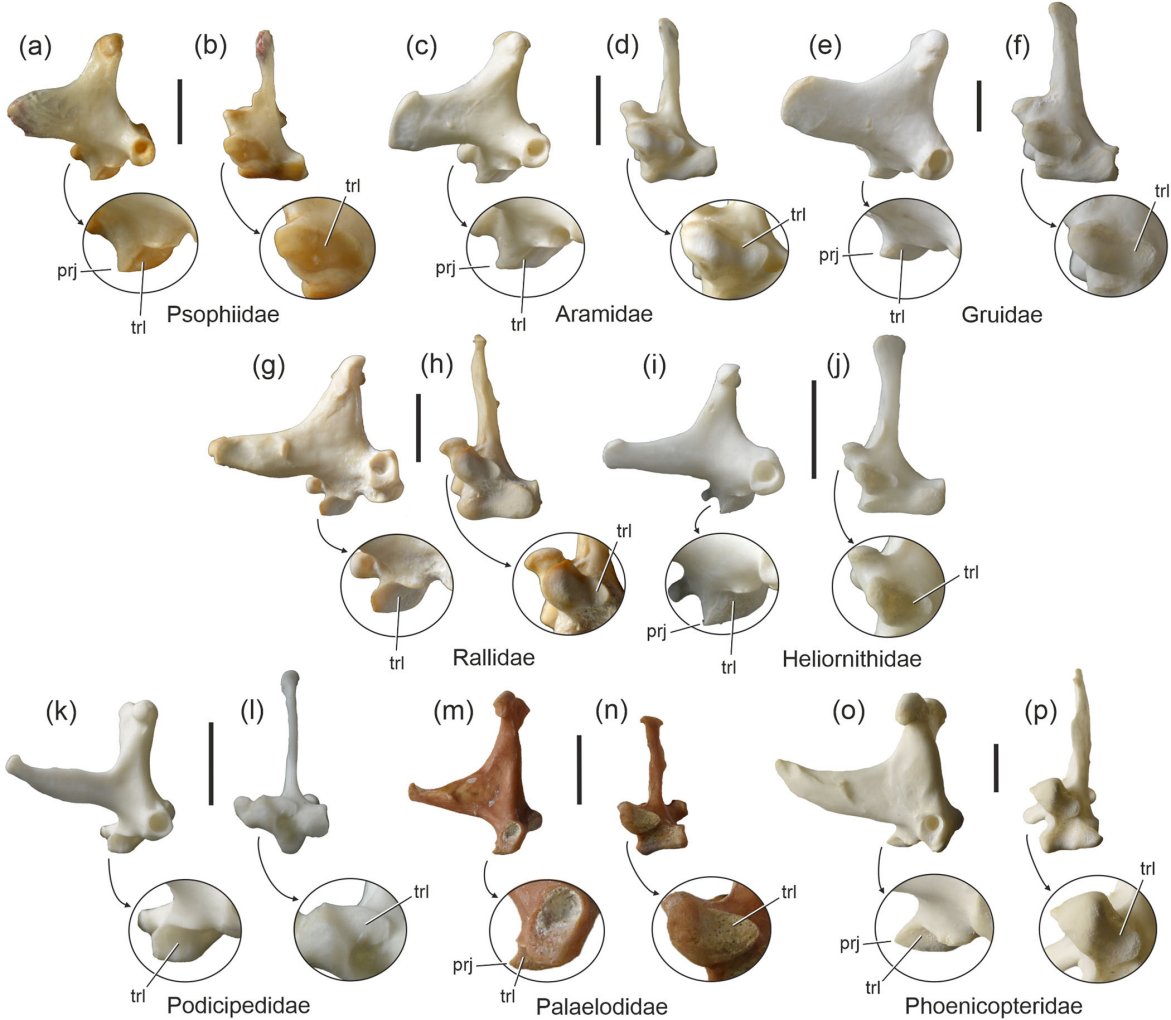
Left quadrate of the Gruiformes (a‒j) and Mirandornithes (k‒p) in lateral (left) and ventral (right) view; the arrows indicate enlarged details of the condylus medialis. (a, b) *Psophia crepitans* (Psophiidae; SMF 2374; right side, mirrored). (c, d) *Aramus guarauna* (Aramidae; SMF 9970). (e, f) *Balearica pavonina* (Gruidae; SMF 13825). (g, h) *Porphyrio poliocephalus* (Rallidae; SMF 11908). (i, j) *Podica senegalensis* (Heliornithidae; SMF 11877). (k, l) *Podiceps cristatus* (Podicipediformes; SMF 14215). (m, n) the early Miocene stem group phoenicopteriform *Palaelodus ambiguus* (Palaelodidae; GPIM Op 395). (o, p) *Phoenicopterus chilensis* (Phoenicopteridae; SMF 14937). Abbreviations: prj, pointed projection of condylus medialis (see text); trl, trochlea lateralis. The scale bar equals 5 mm.

The quadrate of all Gruiformes, that is, all Psophiidae (Figure 4a,b), Aramidae (Figure 4c,d), Gruidae (Figure 4e,f), Sarothruridae, Rallidae (Figure 4g,h), and Heliornithidae (Figure 4i,j), bears a distinct trochlea lateralis. The condylus medialis forms a rostrally directed pointed projection (Figure 4a,c,e,i).

### 3.4 Mirandornithes

This clade includes the Podicipediformes (Figure 4k,l) and Phoenicopteriformes (Figure 4o,p). The condylus medialis of the quadrate of the representatives of both taxa has a trochlea lateralis; this feature is also present in the Palaelodidae (Figure 4m,n), which are mid‐Cenozoic stem group representatives of the Phoenicopteriformes (Mayr 2015a). As in the Gruiformes, the condylus medialis of the Phoenicopteriformes forms a rostrally directed, pointed projection (Figure 4m–p).

### 3.5 Charadriiformes

**FIGURE 5:**
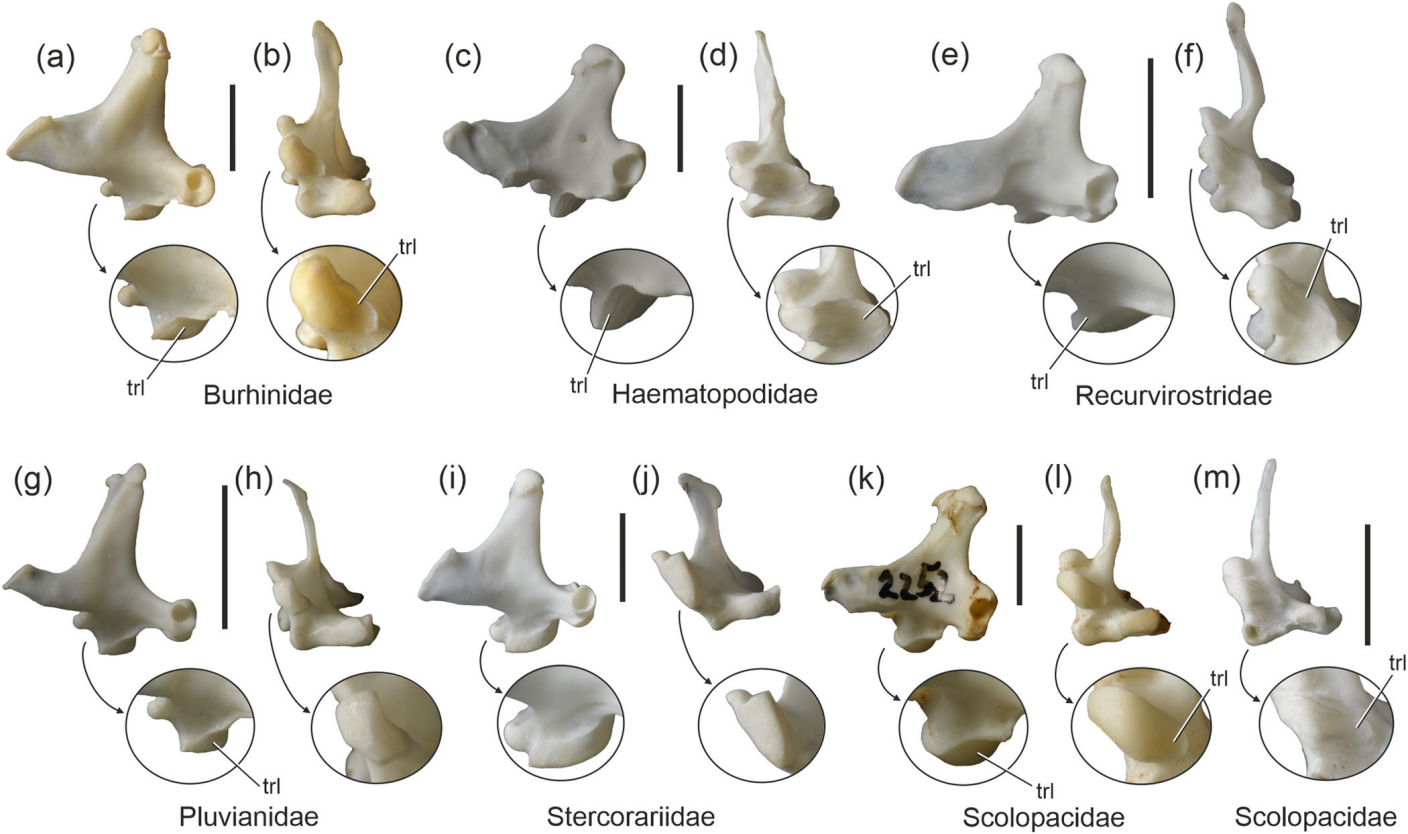
Left quadrate of the Charadriiformes in lateral (left) and ventral (right) view; the arrows indicate enlarged details of the condylus medialis. (a, b) *Burhinus senegalensis* (Burhinidae; SMF 11199). (c, d) *Haematopus ostralegus* (Haematopodidae; SMF 13725). (e, f) *Recurvirostra avosetta* (Recurvirostridae; SMF 14488). (g), (h) *Pluvianus aegyptius* (Pluvianidae; SMF 13544). (i, j) *Stercorarius parasiticus* (Stercorariidae; SMF 13475), which lacks a trochlea lateralis. (k, l) *Numenius arquata* (Scolopacidae; SMF 2252). (m), *Gallinago stenura* (Scolopacidae; SMF 14115 [only ventral view shown]). Abbreviation: trl, trochlea lateralis. The scale bar equals 5 mm.

The Charadriiformes fall into three major subclades termed Charadrii, Scolopaci, and Lari. A trochlea lateralis is present in all representatives of the Charadrii, that is, the Charadriidae, Burhinidae (Figure 5a,b), Haematopodidae (Figure 5c,d), Recurvirostridae (Figure 5e,f), Pluvianidae (Figure 5g,h), and Chionidae; skeletons of *Pluvianellus* (Pluvianellidae) and *Ibidorhyncha* (Ibidorhynchidae) were not available for study, but the concavity was scored as present by Strauch (1978: character 11).

Within the Scolopaci, a trochlea lateralis is found in *Numenius* (Figure 5k,l; erroneously scored absent by Strauch 1978 and Mayr 2011b), which is the sister taxon of other Scolopacidae (Gibson and Baker 2012). A shallow and less distinct trochlea lateralis occurs in *Gallinago* (Figure 5m), *Limosa*, and *Lymnocryptes*. The concavity is absent in *Tringa*, *Calidris*, *Scolopax*, *Xenus*, *Actitis*, *Arenaria*, and *Philomachus*.

Concerning the Lari, Strauch (1978: character 11) erroneously considered a trochlea lateralis to be present in the Laridae, Stercorariidae, Rynchopidae, and Dromadidae. Bock (1960) likewise noted the presence of a trochlea lateralis in the Stercorariidae, but this observation cannot be confirmed and all Stercorariidae lack a trochlea lateralis (Figure 5i,j).

The concavity is well developed in most individuals of *Alca torda* (Alcidae). The condition in this species is notable, because *A. torda* shows individual variation concerning the feature, which is absent in some individuals (Figure 1g–j); all of these were adults as indicated by the closure of cranial sutures, and a sex‐specific variation could not be found. The condylus medialis exhibits a weak trochlea lateralis in *Uria aalge*, whereas it forms an essentially flat surface in *Synthliboramphus antiquus*, *Brachyramphus marmoratus*, *Alle alle*, *Cepphus grylle*, *Fratercula corniculata*, and *F. cirrhata*.

It is not possible to unambiguously determine whether a trochlea lateralis was part of the ground plan, or stem species pattern (ancestral morphology), of the Charadriiformes, because the Charadrii (all of which have the lateral trochlea) are the sister taxon of a clade formed by the Scolopaci and Lari (e.g., Prum et al. 2015; Kuhl et al. 2021). As detailed above, in the Lari and Scolopaci a trochlea lateralis is only present in some Alcidae and Scolopacidae, respectively. However, in most Lari and Scolopaci, the surface of the condylus medialis is not evenly convex but forms a ventral edge that delimits a flat lateral surface medial facet from the ventral facet. This condition—rather than a convex condylus medialis—is likely to be plesiomorphic for charadriiforms and may have favored the convergent origin of a trochlea lateralis in the Charadrii, Scolopaci, and Lari.

### 3.6 Telluraves

**FIGURE 6:**
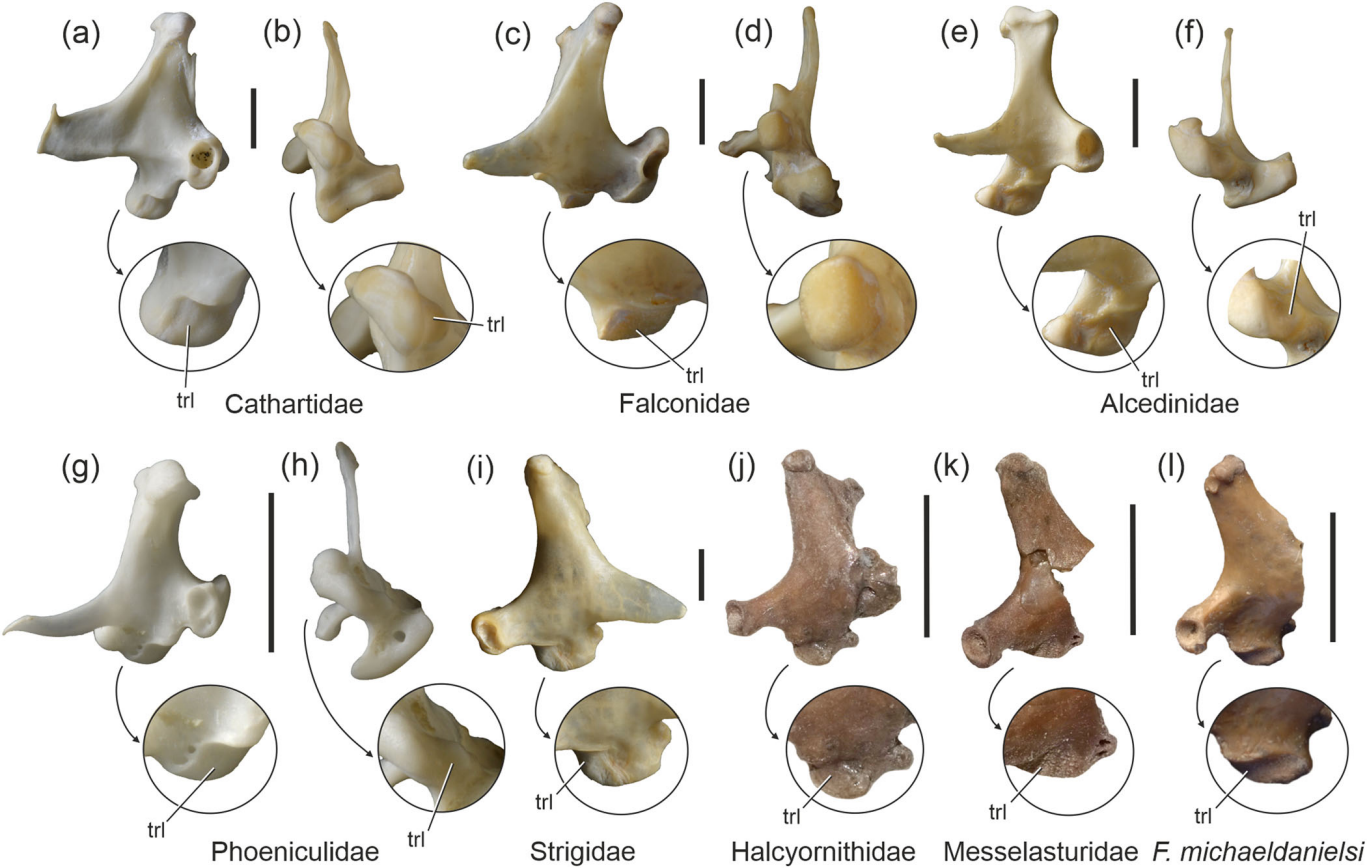
Quadrates of extant and fossil representatives of the Telluraves with a trochlea lateralis; left quadrates in lateral and ventral view (a‒h) and right quadrates in lateral views (i‒l), the arrows indicate enlarged details of the condylus medialis. (a, b) *Coragyps atratus* (Cathartidae; SMF 12266). (c, d) *Caracara plancus* (Falconidae; SMF 6441). (e, f) *Ceryle maxima* (Alcedinidae; SMF 13396). (g, h) *Phoeniculus purpureus* (Phoeniculidae; 12565). (i) *Bubo bubo* (Strigidae; SMF 2621; right side, mirrored). (j) *Pulchrapollia* sp. from the early Eocene London Clay (Halcyornithidae; NMS.Z.2021.40.66). (k) *Tynskya crassitarsus* from the London Clay (Messelasturidae; NMS.Z.2021.40.77). (l) *Fluvioviridavis michaeldanielsi* from the London Clay (Fluvioviridavidae; NMS.Z.2021.40.168). Abbreviation: trl, trochlea lateralis. The scale bar equals 5 mm.

A trochlea lateralis is rarely present in the Telluraves. It is well developed in the Cathartidae (Figure 6a,b) but absent in most other Accipitriformes (in the accipitrid *Harpia harpya* a shallow trochlea lateralis is present). Some Falconidae also exhibit a small trochlea lateralis (Figure 6c,d). In the Strigidae, the condylus medialis exhibits a shallow lateral facet (Fig. 6i), which is weakly developed in the tytonid taxon *Phodilus* and absent in *Tyto* (Tytonidae).

A trochlea lateralis is present in the Upupiformes, being better defined in the Phoeniculidae than in the Upupidae (Figure 6g,h; see also Elzanowski and Boles 2015). Furthermore, a shallow trochlea lateralis is present in the Alcedinidae (see also Elzanowski and Boles 2015), where it is best developed in the taxon *Ceryle* (Figure 6e,f).

According to Bock (1960), the Otididae also exhibit a trochlea lateralis. However, this is only a shallow dimple in some taxa (*Ardeotis*, *Neotis*, *Lophotis*) and absent in others.

## 4 Discussion

The trochlea lateralis of the medial condyle of the quadrate occurs in birds with different bill morphologies and feeding ecologies, and its functional significance remains elusive. A concave facet is often found in long‐beaked species with a rostrally tapering beak, but it is also present in some relatively short‐beaked birds (e.g., Psophiidae, Burhinidae, and Gruidae). The feature occurs in species with holorhinal (e.g., Psophiidae, Burhinidae) and schizorhinal (e.g., Gruidae, Recurvirostridae) nostrils, and in species with (e.g., Threskiornithidae, Recurvirostridae) and without (e.g., Gruiformes, non‐threskiornithid Aequornithes) basipterygoid processes. This diversity of morphologies impedes a straightforward correlation with cranial kinesis and feeding adaptations, and in some taxa the occurrence of a trochlea lateralis more likely has a phylogenetic (shared common ancestry) rather than a functional origin.

Bock (1960: 33) noted that “the jaw articulation of birds is frequently subject to sudden, powerful shocks or to asymmetrical forces, and hence is exposed to the danger of being disarticulated. The problem is, therefore, whether the quadrate hinge in all birds can withstand these strong and possibly disarticulating forces on the mandible or is additional support of the mandible needed in some groups.” The trochlea lateralis articulates with the tuberculum intercotylare of the mandible and certainly contributes to stabilizing the quadratomandibular joint. The morphology may be particularly advantageous in long‐beaked birds, but its exact functional significance still needs to be determined.

Like many other osteological characters, the occurrence of a trochlea lateralis exhibits homoplasy within Neoaves and the feature is found in several taxa, which were shown to be only distantly related in all past and current analyses, such as the Cathartidae and Upupiformes. Its distribution within the Charadriiformes likewise exhibits homoplasy, and the individual variation within *Alca torda* (Figure 1g–j) is particularly notable.

However, the occurrence of a mediolaterally expansive trochlea lateralis in the ground plan of four higher‐level clades, which proved difficult to place in sequence‐based analyses but were recovered as closely related in some of these studies, is of potential phylogenetic significance. These clades are the Aequornithes, Phaethontimorphae, Gruiformes, and Mirandornithes.

All current analyses of nuclear gene sequences support a sister group relationship between the Aequornithes and the Phaethontimorphae (Jarvis et al. 2014; Prum et al. 2015; Kuhl et al. 2021; Stiller et al. 2024), and the clade including both taxa was termed Phaethoquornithes by Sangster et al. (2022). If this clade reflects the true interrelationships of the Phaethontiformes and Eurypygiformes (see Mayr 2022: 118 for a critique), a trochlea lateralis is likely to have been part of the ground plan of the Phaethoquornithes.

Molecular data do not congruently resolve the affinities of the Phaethoquornithes, Gruiformes, and Mirandornithes relative to each other, and there are no two studies, which yielded identical tree topologies. However, a clade ((Phaethoquornithes + Mirandornithes) + Gruiformes) was supported by a recent analysis of Stiller et al. (2024: extended data, figure 4), which only included exon sequences. This clade was not obtained in the analysis of the full genomic data, which resulted in a sister group relationship between the clades (Phaethoquornithes + Strisores) and ((Gruiformes + Charadriiformes) + Opisthocomiformes); Mirandornithes were recovered at the base of Neoaves.

Other analyses showed different results, but do not congruently resolve the affinities of the Mirandornithes and Gruiformes. Earlier studies found a “Metaves” clade, which is not supported by current analyses and included the Mirandornithes and various other taxa, but not the Aequornithes and Gruiformes (Ericson et al. 2006; Hackett et al. 2008). When the β‐fibrinogen gene was excluded—which accounted for the “Metaves” clade—, the analysis of Ericson et al. (2006: fig. ESM‐6) supported a clade including the Aequornithes, Phaethontiformes, Charadriiformes, Gruiformes, Mirandornithes, Mesitornithiformes, Otidiformes, Musophagiformes, and Opisthocomiformes.

The analysis of Jarvis et al. (2014) found the Phaethoquornithes to be the sister taxon of the Telluraves, with a clade (Opisthocomiformes + (Gruiformes + Charadriiformes)) branching next; Mirandornithes were placed in a widely separated clade together with the Columbiformes, Pterocliformes, and Mesitornithiformes. An analysis of Kuhl et al. (2021) likewise supported a sister group relationship between the Phaethoquornithes and Telluraves; Mirandornithes were recovered as the sister taxon of all other Neoaves, and Gruiformes and Charadriiformes resulted as sister taxa in a distant position to the clade formed by Phaethoquornithes and Telluraves.

The study of Prum et al. (2015) recovered the Phaethoquornithes as the sister taxon of the Opisthocomiformes and Telluraves, with a clade including the Charadriiformes and Mirandornithes diverging next, followed by the Gruiformes. By contrast, an analysis of Wu et al. (2024) supported a clade including the Phaethoquornithes, Opisthocomiformes, and Mirandornithes, and this latter clade formed the sister taxon of a clade including the Charadriiformes, Gruiformes, and Strisores.

From a morphological point of view, there exists no evidence for close affinities between the Aequornithes (or Phaethoquornithes) and the Telluraves, Opisthocomiformes, or Strisores. The quadrates of the Opisthocomiformes and Strisores, and that of most (see above) representatives of the Telluraves lack a trochlea lateralis, and molecular phylogenies do not show congruent results concerning the interrelationships of these taxa. The hoatzin is an oddball taxon, which proved notoriously difficult to place phylogenetically (e.g., Wang et al. 2021), and the Strisores are likewise recovered in disparate positions in sequence‐based analyses (compare, e.g., Prum et al. 2015; Kuhl et al. 2021, and Stiller et al. 2024).

Even though molecular sequence data do not conclusively resolve the interrelationships of the Phaethoquornithes, Gruiformes, and Mirandornithes, these three taxa are shown to be closely related in some studies (Prum et al. 2015; Wu et al. 2024) and in one analysis of Stiller et al. (2024: extended data, figure 4) they form a clade. Therefore, there exists some molecular and morphological evidence that these three taxa may actually form a previously unrecognized clade. Most Mirandornithes and Gruiformes also share a rostrocaudally narrow condylus medialis, which forms a rostrally directed, pointed projection (Figure 4), and close affinities between both taxa were suggested by Mayr (2014, 2015a) based on features other than the morphology of the quadrate. Certainly, it would be premature to attach too much importance to the phylogenetic significance of the trochlea lateralis in the Aequornithes, Phaethontimorphae, Gruiformes, and Mirandornithes. However, in light of the conflicting evidence for a phylogenetic placement of these taxa, the feature is one of the few candidates for a well‐defined morphological character that may contribute to a better understanding of avian higher‐level phylogeny.

The morphology of the condylus medialis of the quadrate is also of potential significance for the assessment of the affinities of fossil species. In addition to a few taxa, where a trochlea lateralis is expected owing to their presumed phylogenetic position, such as the stem group gaviiform *Nasidytes* (Mayr and Kitchener 2022) and the stem group threskiornithid *Rhynchaeites* (Mayr and Kitchener 2023), this feature also occurs in a few Paleogene fossil birds of uncertain phylogenetic affinities. For example, it is present in a small quadrate from the early Eocene of Australia (Elzanowski and Boles 2015) and was one of the features why the fossil was likened to the Upupiformes.

The presence of a trochlea lateralis supports the presumed (see Mayr and Kitchener 2024) sister group relationship between the early/middle Eocene Halcyornithidae (Figure 6j) and Messelasturidae (Figure 6k), which was recovered in some analyses (e.g., Mayr 2011c), but not in others (e.g., Mayr 2015b). These two taxa are part of Telluraves, and in some analyses they resulted within Psittacopasseres, the clade including the Psittaciformes and Passeriformes. However, the higher‐level affinities of messelasturids and halcyornithids are not well established and some features are shared with falconiforms and strigiforms(Mayr 2015b, 2022) to which the quadrate likewise shows a resemblance. A trochlea lateralis is also found in the quadrate of a bird from the early Eocene of the London Clay, which was described as *Fluvioviridavis michaeldanielsi* by Mayr and Kitchener (in press) (Figure 6l). The affinities of *Fluvioviridavis* are poorly resolved. The taxon resulted as the sister taxon of the Podargiformes (Nesbitt et al. 2011) or Steatornithiformes (Chen et al. 2019), but Mayr and Kitchener (in press) listed the trochlea lateralis of the quadrate and other features, in which *Fluvioviridavis* differs from all extant Strisores. Actually, the quadrate of *F. michaeldanielsi* is similar to that of the Halcyornithidae and Messelasturidae, and further research is needed to establish the actual affinities of this species. Finally, the absence of a trochlea lateralis shows the taxon *Walbeckornis* to be outside Gruiformes and refutes the hypothesis that it may be more closely related to the gruiform Messelornithidae (Mayr and Kitchener 2025).

## Author Contributions

**Gerald Mayr:** conceptualization, investigation, methodology, validation, visualization, formal analysis, writing – review and editing, writing – original draft.

## Conflicts of Interest

The author declares no conflicts of interest.

## 1 Peer Review

The peer review history for this article is available at https://www.webofscience.com/api/gateway/wos/peer-review/10.1002/jmor.70070.

## Data Availability

All data are included in the article. Data sharing is not applicable to this article as no further new data were created or analyzed in this study.
